# Phase-adaptive brain stimulation of striatal D1 medium spiny neurons in dopamine-depleted mice

**DOI:** 10.1038/s41598-022-26347-z

**Published:** 2022-12-16

**Authors:** Youngcho Kim, Dennis Jung, Mayu Oya, Morgan Kennedy, Tomas Lence, Stephanie L. Alberico, Nandakumar S. Narayanan

**Affiliations:** 1 Department of Neurology, University of Iowa, 169 Newton Road, Pappajohn Biomedical Discovery Building-1336, Iowa City, IA 52242 USA; 2 University of Rochester Medical Center, Rochester, New York, NY 14642 USA; 3 Carver College of Medicine, University of Iowa, Iowa City, IA 52242 USA; 4 University of Minnesota, Minneapolis, MN 55455 USA

**Keywords:** Basal ganglia, Brain-machine interface

## Abstract

Brain rhythms are strongly linked with behavior, and abnormal rhythms can signify pathophysiology. For instance, the basal ganglia exhibit a wide range of low-frequency oscillations during movement, but pathological “beta” rhythms at ~ 20 Hz have been observed in Parkinson’s disease (PD) and in PD animal models. All brain rhythms have a frequency, which describes how often they oscillate, and a phase, which describes the precise time that peaks and troughs of brain rhythms occur. Although frequency has been extensively studied, the relevance of phase is unknown, in part because it is difficult to causally manipulate the instantaneous phase of ongoing brain rhythms. Here, we developed a phase-adaptive, real-time, closed-loop algorithm to deliver optogenetic stimulation at a specific phase with millisecond latency. We combined this Phase-Adaptive Brain STimulation (PABST) approach with cell-type-specific optogenetic methods to stimulate basal ganglia networks in dopamine-depleted mice that model motor aspects of human PD. We focused on striatal medium spiny neurons expressing D1-type dopamine receptors because these neurons can facilitate movement. We report three main results. First, we found that our approach delivered PABST within system latencies of 13 ms. Second, we report that closed-loop stimulation powerfully influenced the spike-field coherence of local brain rhythms within the dorsal striatum. Finally, we found that both 4 Hz PABST and 20 Hz PABST improved movement speed, but we found differences between phase only with 4 Hz PABST. These data provide causal evidence that phase is relevant for brain stimulation, which will allow for more precise, targeted, and individualized brain stimulation. Our findings are applicable to a broad range of preclinical brain stimulation approaches and could also inform circuit-specific neuromodulation treatments for human brain disease.

## Introduction

Neuronal activity involves electrical potentials that oscillate over time^1^. Brain rhythms describe voltage oscillations in the time domain. These oscillations are characterized by a frequency, how often they oscillate (Hz), and a phase (radians), the precise time that peaks and troughs of oscillations occur. For instance, brain rhythms with the same frequency and opposite phase might have different voltage depending on the instantaneous phase^2^. The frequency of brain rhythms has been extensively studied^3,4^. However, the phase of brain rhythms may be particularly relevant for brain stimulation in cases where pulses might arrive at the peak of a phase, when voltage is high, or at the trough of a phase, when voltage is low^5,6^. These stimulation pulses might have very different effects dependent on the phase of oscillating brain rhythms. With the advent of clinical brain stimulation for Parkinson’s disease (PD) and other neuropsychiatric diseases^7–9^, determining the significance of brain rhythm phase is critical to future efforts of developing brain stimulation therapies for human brain disease.

We explored oscillation frequency and phase in forebrain networks in the dorsal striatum, a key brain structure for motor control. The dorsal striatum has marked movement-related low-frequency oscillations^10–13^. In addition, dorsal striatal beta rhythms at ~ 20 Hz are linked with movement suppression^12,14,15^. We and others have also noted changes in lower frequency rhythms with human PD and in animal models^10,15–19^. Critically, these beta rhythms are pathologically increased in PD and depend on the neurotransmitter, dopamine, which is a key treatment in PD^14,20,21^. Closed-loop brain stimulation, which has been shown to improve motor symptoms of human PD, can be triggered by the amplitude of beta rhythms^22,23^. However, despite insights that the phase of different rhythms can be coupled^24,25^, it is unclear precisely what role phase is playing in dorsal striatal brain rhythms. A key barrier lies in the difficulty to detect the phase of brain rhythms in real-time, and in the even more difficult challenge to deliver brain stimulation to manipulate these rhythms with closed-loop stimulation. Indeed, causally studying the phase of brain rhythms requires perturbing brain networks at temporally precise moments on the scale of milliseconds to target specific phases (peak—0 radians, or trough—pi radians) of these ongoing rhythms.

Here we developed techniques and algorithms for real-time stimulation with millisecond system latencies between recordings and stimulation. This system enabled us to test the hypothesis that Phase-Adaptive Brain STimulation (PABST) of striatal brain rhythms impacts head movement. We tested this idea in dopamine-depleted mice, which have well-described movement impairments and marked changes in striatal brain rhythms that can be isolated and targeted by PABST^10,19,26^. In these mice, we delivered closed-loop optogenetic stimulation of D1-dopamine expressing striatal medium spiny neurons (D1-MSNs). We report three results: (1) Our PABST system has sufficiently low latency (13 ms) to deliver laser pulses in phase with ongoing rhythms; (2) phase-specific striatal stimulation powerfully affects spike-field coherence in the dorsal striatum, but not local field potentials (LFPs); and (3) while 4 Hz PABST and 20 Hz PABST improved head movement speed, we found phase differences only at 4 Hz. These data suggest that phase is important for 4 Hz striatal oscillations. These findings, although in a limited context, are relevant for understanding forebrain brain rhythms, for refining preclinical brain stimulation efforts, and for delivering optimized and individualized clinical brain stimulation.

## Methods

### Mice

Eleven D1 dopamine receptor (D1DR)-Cre mice (Drd1a-cre+; derived from Gensat strain EY262; aged 3 months; 25–32 g; D1DR-Cre) were used in this study. Mice genetics were verified by genotyping using primers for the D1-Cre recombinase transgene (D1-Cre-F: 5′-AGG GGC TGG GTG GTG AGT GAT TG; D1-Cre-R: CGC CGC ATA ACC AGT GAA ACA GC-3′). All procedures were approved by the Institutional Animal Care and Use Committee (#0062039) at the University of Iowa, in accordance with the National Institutes of Health Guide for the Care and Use of Laboratory Animals and the ARRIVE guidelines.

### Dopamine-depletion and ChR2 expression

As in our previous work, we used an adeno-associated vector (AAV) construct with floxed-inverted channelrhodopsin (AAV-ChR2), along with mCherry (University of North Carolina at Chapel Hill (UNC) Vector Core; AAV5-EF1a-DIO-hChR2(H134R)-mCherry) to stimulate neurons expressing D1DRs^27,28^. In a single surgery, we injected mice with AAV-ChR2 (0.5 μL of approximately ≥ 1 × 10^13^ viral genomes/mL) into the dorsal striatum (AP + 0.5/ML − 1.5/DV − 3.0) and immediately placed an optical fiber cannula (200 μm core, 0.22 NA, Doric Lenses) at the same coordinates as the injection site. We also unilaterally injected the neurotoxin, 6-hydroxydopamine (6-OHDA; 2.5 μL with 2 μg/μL), into the dorsal striatum in two injection locations (AP + 0.4/ML − 1.8/DV − 3.5 and AP + 1.1/ML − 2.0/DV − 3.5) to deplete dopamine; of note, this manipulation consistently kills nigrostriatal dopamine terminals and produces marked changes in striatal field potentials^10,19^. Three additional sham mice had an identical surgery with saline injected into the dorsal striatum instead of 6-OHDA; The extent of ChR2 expression is quantified using mCherry expression. All other experimental procedures were identical. All stimulation experiments were performed after > 4 weeks of recovery and ChR2 expression.

### Phase-adaptive brain stimulation (PABST)

**Figure 1:**
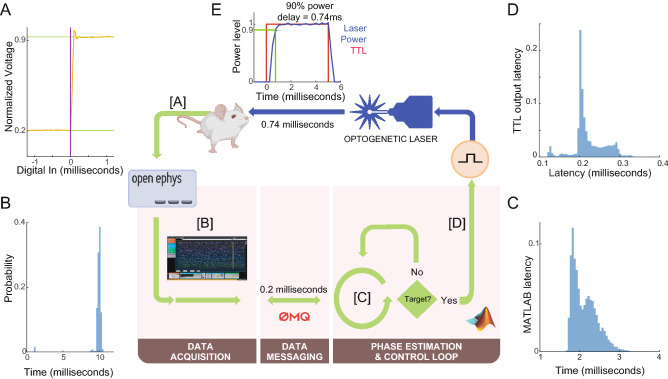
Phase-Adaptive Brain STimulation (PABST). (**A**) Brain recordings from an implanted mouse sampled at 24,000 samples/second are acquired via Open Ephys, and (**B**) buffered with a delay of ~ 10 ms. (**C**) The data is then transferred to a computer running MATLAB for phase estimation and closed-loop control with ~ 2.5 ms latency. After phase estimation and stimulation decisions are made, (**D**) a TTL is sent to an optogenetic laser, which (**E**) powers up over 0.74 ms, delivering a 5-ms pulse of 473-nm light.

For PABST, we implanted 16-channel microwire optrodes (Microprobes) in the dorsal striatum (AP + 0.5/ML − 1.5/DV − 3.0; Fig. 1). We recorded LFPs across 16 channels using an Open Ephys system. The raw signal was amplified with a total gain of 198X, high-pass filtered at 0.1 Hz, and recorded with 16-bit resolution at a 30 k-Hz sampling rate.

To estimate phase, LFPs were streamed from the Open Ephys system to a notebook computer (Dell) via a zeroMQ data messaging system. Real-time analysis was performed in MATLAB; data were buffered over ~ 10 ms. Wavelet-based frequency and phase estimation were performed using a complex Morelet. Instantaneous frequency and phase were calculated using wavelets and predicted for 50 ms in the future using linear estimation.

During closed-loop experiments, D1DR+ mice with optical cannula were connected to the optical patch cable through a zirconia ferrule (Doric Lenses) without anesthesia. Light was generated using a 473-nm diode-pumped solid-state (DPSS) laser source (OEM Laser Systems), and an optical rotary joint (Doric Lenses) was used to facilitate animal rotation. We used a 5 ms pulse width, which was controlled via TTL signals sent through a microcontroller controlled by a computer running PABST. Before each experiment, the power output of the laser was adjusted to ~ 10 milliWatts the fiber tip. Power measurements verified that the laser reached 90% power within 0.74 ms of TTL triggers and maintained ~ 10 milliWatts with < 5% error and stimulated spiking activity (Figure S1). Of note, we delivered 4 Hz and 20 Hz PABST in the same animals with a randomized session order, making it possible to compare effects of PABST within mice with identical electrode locations and ChR2 expression.

### Motion tracking

We captured motion using a 3-D motion-tracking system (OptiTrack); we have previously used this system to track mouse movement in detail^10,19^. Briefly, we implanted two 4-mm infrared-reflective spheres attached to the recording headstage in the anterior–posterior dimension. Four infrared cameras recorded the X (right-left), Y (forward-back), and Z (up-down) coordinates of the mouse’s head at 120 frames/per second (frames/s) to track head position^10^. Automated computer tracking data were synchronized with a video camera at 30 frames/s and neurophysiological recording hardware.

### Histology

ChR2 expression and electrode localization were confirmed by immunohistochemistry. Briefly, mice were deeply anesthetized with ketamine (100 mg/kg) and xylazine (10 mg/kg) and intracardially perfused with ice-cold 4% paraformaldehyde. Brains were post-fixed in 4% paraformaldehyde overnight and immersed in 30% sucrose until the brains sank. Brains were sectioned (40 or 50 μm) with a cryostat (Leica) and stored in PBS. Immunostaining procedures were performed with free-floating brain sections. Primary antibodies to tyrosine hydroxylase (rabbit anti-TH; Millipore −MAB152; 1:500–1000) were incubated overnight at 4 °C. Sections were visualized with Alexa Fluor fluorescent secondary antibodies goat anti-rat IgG Alexa 568, Thermo Fisher Scientific; 1:1000) and matched with the host primary by incubating 2 h at room temperature. Images were captured on an Olympus VS120 microscope.

### Statistics

All procedures were reviewed by the Biostatistics, Epidemiology, and Research Design Core within the University of Iowa Institute for Clinical and Translational Science. To avoid making any distributional assumptions about the data, continuous measures were reported as medians and inter-quartile ranges. Testing for significant differences followed the non-parametric Wilcoxon rank sum test.

## Results

We tested the hypothesis that the phase-adaptive brain stimulation (PABST) of dorsal striatal brain rhythms impacts behavior. We developed a real-time, closed-loop algorithm to calculate the ongoing instantaneous phase of brain rhythms from striatal LFPs to effect PABST. We streamed striatal LFPs to a dedicated computer for real-time preprocessing, filtering, and phase calculation in MATLAB. The sources of latency in these calculations were: (A) headstage to Open Ephys acquisition system latency: < 0.1 ms (Fig. 1A); (B) input buffer latencies (required for time–frequency analyses): 10.00 (9.87–10.10) milliseconds [all values median (Q1–Q3); Fig. 1B); (C) calculation latency: 2.05 (1.86–2.32) milliseconds (Fig. 1C); and (D) Open Ephys to MATLAB latency: 0.21 (0.20–0.24)] milliseconds (Fig. 1D). During optogenetic brain stimulation, the 473-nm laser achieved 90% power at 0.74 ms with 5 ms pulse width (Fig. 1E). Although it is difficult to simultaneously measure latencies and deliver stimulation, we estimated the total median latency as 12.98 ms. Because this latency is less than the 50 ms period of 20 Hz rhythms, these data indicate that this system latency is compatible with closed-loop PABST targeting dorsal striatal beta rhythms.

**Figure 2:**
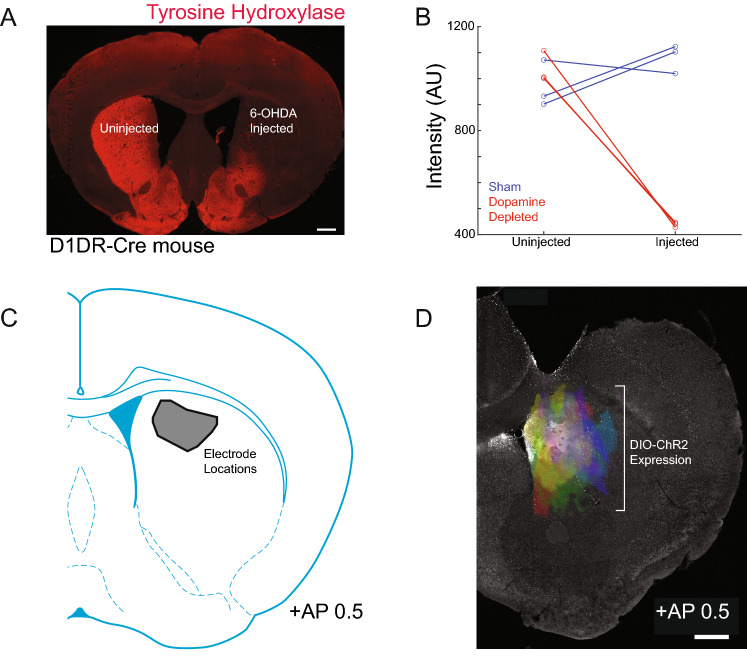
Dopamine depletion, recording, and viral expression: (**A**) we depleted dopamine by injecting 6-OHDA in the dorsal striatum of D1DR-Cre mice. The terminals of dopamine neurons expressing tyrosine hydroxylase are stained in red. Scale bar 500 μm. (**B**) Tyrosine hydroxylase fluorescence was reliably decreased in 6-OHDA dopamine depleted animals (red) compared to sham animals that underwent identical procedures except with saline injected into the dorsal striatum (blue). (**C**) Electrode locations in the dorsomedial striatum. (**D**) Viral DIO-ChR2 expression in D1DR-Cre mice; each color is an individual animal, aligned to a background image from a single animal. Data in (**B**–**D**) from 3 dopamine-depleted and 3 sham animals. Scale bar 500 μm.

We harnessed PABST to deliver closed-loop, real-time brain stimulation in the dorsal striatum. We depleted dopamine using the neurotoxin 6-OHDA, which decreased striatal dopaminergic terminals (Fig. 2A,B). This manipulation reliably impairs movement and changes striatal oscillations^10,19,26^. We implanted recording electrodes in the mouse dorsomedial striatum (Fig. 2C) and virally-expressed ChR2+ in D1-MSNs (Fig. 2D).

**Figure 3:**
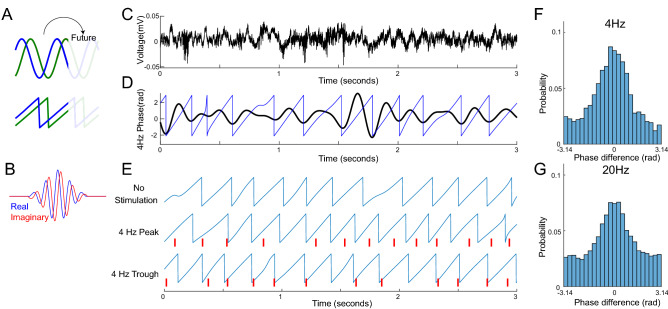
Phase accuracy of PABST. (**A**) To deliver closed-loop stimulation in a system with lag, we estimated oscillatory phase at future time points. (**B**) We used a complex Morlet to estimate phase and frequency. (**C**) Example of raw striatal local field potential (LFP; 3 s), (**D**) the same signal filtered at 4–8 Hz (black), and the estimated 4 Hz phase (blue). (**E**) Example trials without stimulation (top), with PABST at the peak (0 rads; middle panel) or at the trough (pi rads; bottom panel; red lines indicate PABST delivery). (**F**) Phase accuracy for all sessions from 6 mice at 4 Hz and at (**G**) 20 Hz PABST.

We estimated the phase-accuracy of PABST in targeting 4 Hz and 20 Hz dorsal striatal brain rhythms. Because all real-time brain stimulation systems have some lag, they must predict the phase at some point in the future, even if it is a few milliseconds (Fig. 3A). We estimated the phase of striatal LFPs (Fig. 3B–D) and delivered real-time, closed-loop stimulation pulses targeting 4 Hz and 20 Hz rhythms (Fig. 3E). We found that at 4 Hz the system produced an average phase difference of − 0.01 (− 0.90 to 0.82; Fig. 3F), and at 20 Hz the system produced an average phase difference of 0.10 (− 0.93 to 1.11; Fig. 3G). These data indicate that we can deliver phase-adaptive brain stimulation to striatal LFPs at 4 Hz and 20 Hz.

**Figure 4:**
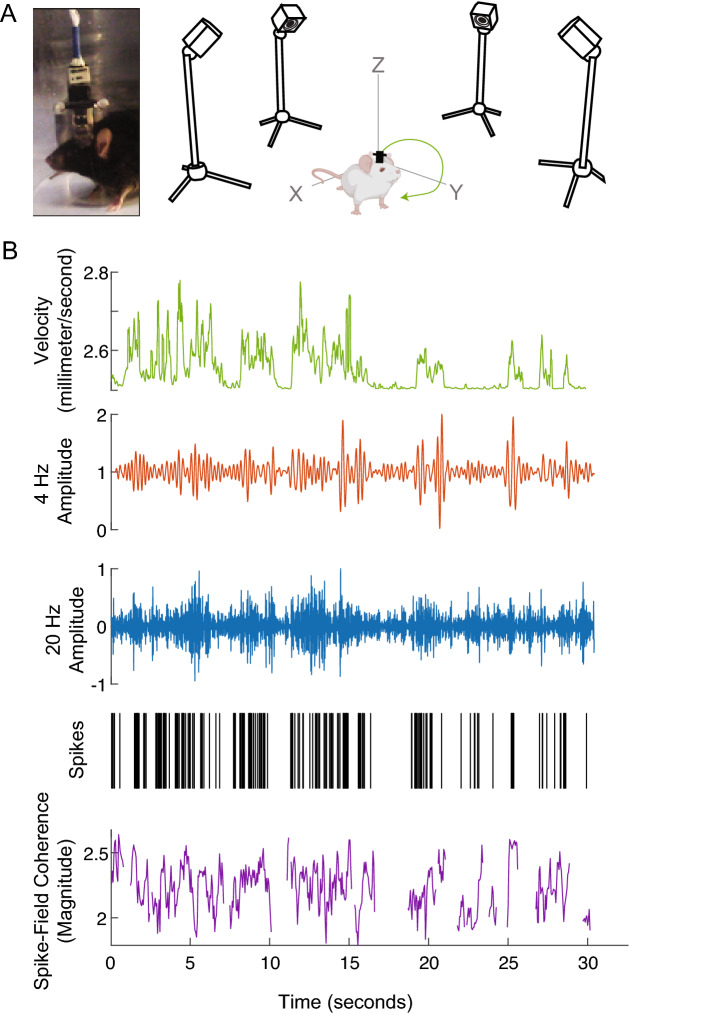
Striatal dynamics change with movement. (**A**) We tracked head movement using infrared-reflective spheres and an array of four cameras (OptiTrack). (**B**) We found that while animals moved, 4 Hz filtered (red) and 20 Hz filtered (blue) local field potentials (LFPs) were dynamic, as was the spike rate of medium spiny neurons (MSN; black) and 4 Hz spike-field coherence (purple; note that this value sometimes became indeterminate). Data from one mouse.

We tracked the position of mice at ~ 10-ms accuracy using an infrared tracking system (Fig. 4A). With this approach, we were able to record from striatal neuronal ensembles and LFPs in dopamine-depleted animals as they moved freely and were able to deliver real-time phase-responsive optogenetic stimulation of D1DR+ neurons (Fig. 4B). Of 358 recorded neurons, 73 had significantly increased firing rate with < 5 ms opto-stimulation latency (Figure S1).

**Figure 5:**
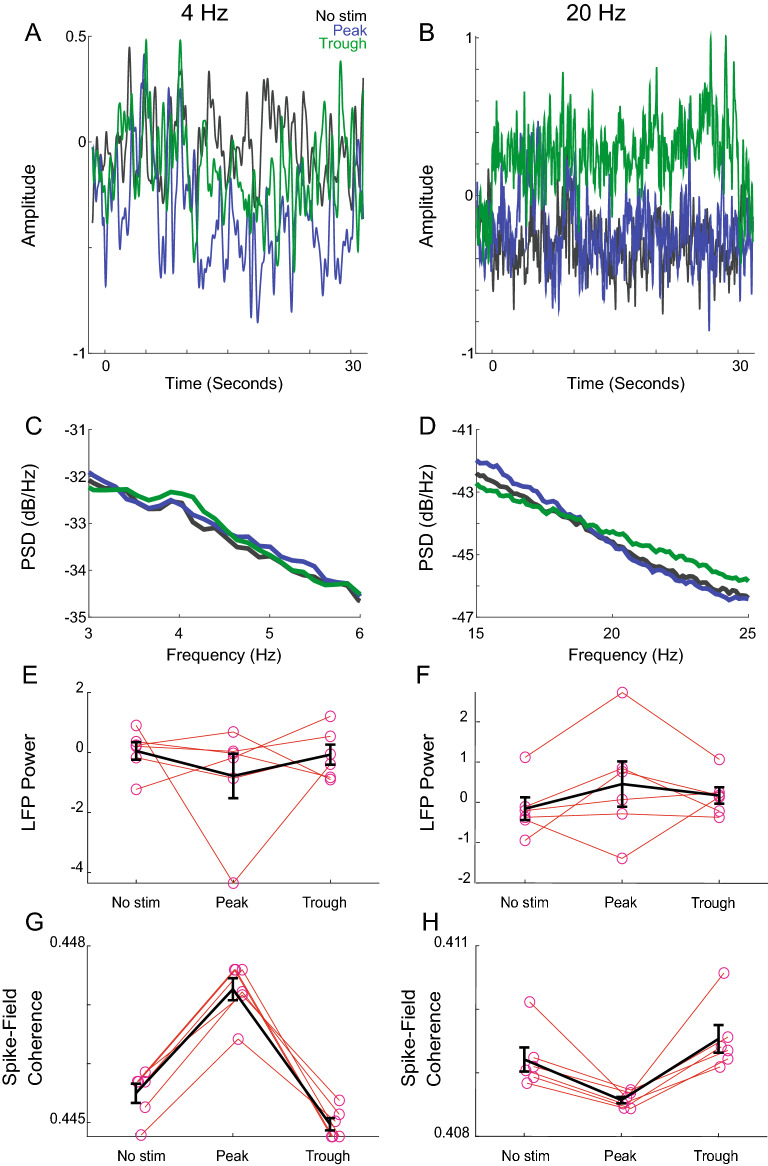
Closed-loop striatal stimulation changes spike-field coherence but not LFPs. (**A**) Raw traces from 4 Hz stimulation sessions during 30-s epochs without no stimulation (No stim; black), with peak PABST (blue), and trough PABST (green). (**B**) Raw traces for 20 Hz stimulation. (**C**) Power spectral density for 4 Hz PABST sessions, and for (**D**) 20 Hz PABST sessions. There were no reliable effects of peak vs. trough closed-loop stimulation on striatal power at (**E**) 4 Hz or at (**F**) 20 Hz. However, we noticed that (**G**) 4 Hz peak-stimulation spike-field coherence was increased relative to trough spike-field coherence, whereas for (**H**) 20 Hz stimulation trough stimulation had a higher spike-field coherence than peak stimulation. Data from six dopamine-depleted mice.

Next, we examined how PABST influenced striatal brain networks. We recorded LFPs and neuronal activity in six mice during 30-s epochs without stimulation with closed-loop stimulation targeting either 4 Hz or 20 Hz peaks. We found that there was no reliable difference in striatal power spectra between peak vs. trough stimulation (Fig. 5A–F). However, we found consistent differences in spike-field coherence with 4 Hz and 20 Hz peak vs. trough stimulation (Spike-field coherence, 4 Hz peak: *p* = 0.005; Cohen’s d = 6.2, Fig. 5G) and (Spike-field coherence, 20 Hz peak: *p* = 0.002; Cohen’s d = 2.5, Fig. 5H). These data suggest that in the dorsal striatum, 4 Hz peak stimulation increased spike-field coherence, whereas 20 Hz peak stimulation decreased spike-field coherence.

**Figure 6:**
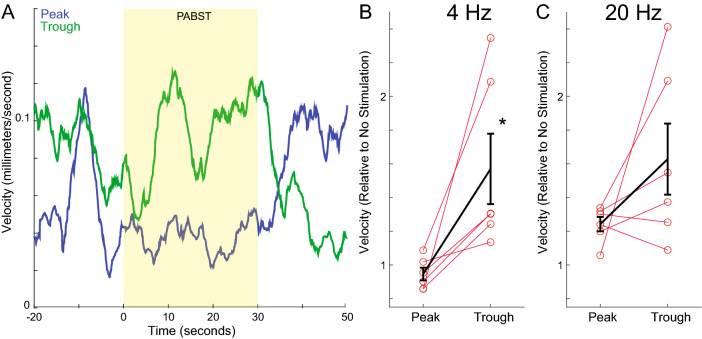
Closed-loop 4 Hz striatal trough stimulation improves movement velocity in dopamine-depleted mice. (**A**) We stimulated D1-expressing medium spiny neurons (D1-MSNs) at the peak or trough of 4 Hz striatal brain rhythms using PABST; example trace from one mouse from an average epoch. (**B**) We found that 4 Hz trough PABST increased head movement velocity compared to 4 Hz peak stimulation, suggesting that 4 Hz phase may be relevant to brain stimulation. (**C**) By contrast, the phase of 20 Hz rhythms did not reliably affect movement velocity. Data from six mice.

Finally, we examined whether PABST influences head movement velocity, as has been shown previously for striatal D1-MSN stimulation^26,29^. We found that stimulation of D1-MSNs in-phase with 4 Hz oscillations at troughs, but not peaks, increased head movement speed (Fig. 6A). Of note, these movements were quite distinct from high-velocity dyskinetic movement observed with high-dose levodopa or with high-intensity optogenetics^10,30^ (Supplemental Video 1). We compared velocity changes in six mice for peak vs. trough PABST stimulation relative to epochs with no stimulation (Fig. 6B,C). Strikingly, we found that 4 Hz trough PABST reliably increased velocity relative to 4 Hz peak PABST [peak 0.93 (0.86–1.02) vs. trough 1.30 (1.24–2.09); *p* = 0.002; Cohen’s d = 1.7; 41% increase over peak stimulation; Fig. 6B]. We did not observe reliable differences for 20 Hz peak vs. trough PABST [peak: 1.27 (1.20–1.32) vs. trough 1.46 (1.25–2.09; *p* = 0.132; Fig. 6C]. However, we note that trough 4 Hz stimulation increased absolute head movement speed relative to no stimulation (*p* = 0.002; Cohen’s d = 1.6; Figure S2), and both peak and trough 20 Hz stimulation increased head movement speed relative to no stimulation (peak 20 Hz; *p* = 0.002; Cohen’s d = 3.3; trough 20 Hz; *p* = 0.002; Cohen’s d = 1.7; Figure S2). Finally, we did not observe velocity improvements identical experiments with phase-random stimulation or in sham animals (Figure S3). Taken together, these data suggest that the phase of 4 Hz PABST is relevant to D1-MSN stimulation of movement velocity. These data provide insight into the nature of D1-MSN firing relative to striatal brain rhythms and could be useful for future brain stimulation technologies.

## Discussion

We interrogated striatal brain rhythm phase using adaptive brain stimulation. We used PABST to deliver optogenetic pulses at the peak phase or trough phase of ongoing striatal brain rhythms. First, we found that the latency of PABST was 13 ms, and that we could deliver phase-accurate stimulation at the peak/trough with 4 Hz and 20 Hz rhythms. Second, PABST targeting D1-MSNs powerfully modulated spike-field coherence of striatal brain rhythms, but not the overall power of striatal brain rhythms. Lastly, while both 4 Hz and 20 Hz improved head movement speed, 4 Hz trough PABST increased head movement velocity by 41% compared to 4 Hz peak PABST. These data directly test the hypothesis that the phase of ongoing brain rhythms is relevant to brain stimulation and provide insight into brain rhythms in the dorsal striatum.

Neural systems are characterized by diverse and complex rhythms^1^. Our work demonstrates a novel, causal role for dorsal striatal brain rhythm phase in movements, albeit in a highly limited context—optogenetic D1-MSN stimulation of the dorsal striatum in dopamine-depleted mice. Our rationale for studying dopamine-depleted mice is that (1) they have readily detected deficits in movement that can be dynamically measured, and (2) dopamine-depletion markedly changes striatal brain rhythms which can be detected by phase-adaptive technologies such as PABST^10,19^. Notably, PABST had few reliable effects in sham-animals with intact dopaminergic circuits (Figure S3). These data suggest that PABST may be effective under certain constraints, and future experiments with different behavioral measures and disease models will help clarify these constraints. However, our findings establish that PABST has the potential to be effective in rodent models of PD.

In the dorsal striatum, bursts of low-frequency rhythms can be powerfully affected by movement^13,31^. Because these rhythms are abnormal in basal ganglia disorders in preclinical models of movement disorders such as PD^10,11,15,17^, and because these rhythms synchronize striatal oscillations^32^, understanding the details of these oscillations is highly relevant for human disease. D1-MSN coherence with these oscillations was not explicitly related to movement, but 4 Hz trough PABST decreased D1-MSN coherence and improved head movement velocity. There may be specific populations of D1-MSNs that are linked with head movement and facilitated by specific spectral aspects of striatal LFPs. Future work will study these head-movement-specific D1-MSNs.

PABST is a new technology, combining off-the-shelf equipment to deliver phase-responsive stimulation with millisecond precision to dynamic striatal networks. This technology could be used, along with other similar techniques^33^ to probe a variety of neuronal oscillations in the context of behavior and disease. Indeed, most preclinical and all clinical brain stimulation are delivered without consideration of these underlying brain rhythms and phase. Closed-loop technologies may be critical to adjusting brain stimulation to ongoing dynamics, so considering brain rhythm frequency and phase might help maximize the efficacy of brain stimulation for a wide range of applications^6,34^.

We found that 4 Hz trough PABST of D1-MSNs is effective for improving head movement velocity. In the context of D1-MSNs, our work is consistent with prior work that has shown that D1-MSN stimulation facilitates movement^26,35^. Our work advances prior stimulation approaches by showing that highly precise 4 Hz trough PABST improved movement velocity in dopamine-depleted animals. It is possible that PABST can have complex effects on striatal networks affecting our results at 20 Hz. Understanding these effects could be important not only for a basic understanding of basal ganglia networks, but also for targeted brain stimulation interventions to improve movement disorders^8,36^. Head movement may not directly correlate with overall body movement, and there may many other movement effects of PABST that more advanced measurements of movement might capture^37^. Current adaptive brain stimulation approaches target the power in frequency-specific bands, whereas we target the phase^22^. While our work is currently a proof-of-concept in preclinical models, PABST has the potential to be more powerful while delivering fewer pulses, and thus more efficient, even when considering the computational requirements of PABST. Future work will investigate PABST’s potential to treat movement disorders.

D1-MSNs have been strongly implicated in dyskinesias^38^. Stimulation of these neurons with higher stimulation frequencies (10–20 Hz) has also been shown to promote dyskinesias^10,39^. We did not observe dyskinesias with stimulation in the present study, but dyskinesias may occur in animals receiving high-dose levodopa or brain stimulation. We have observed 4 Hz spike-field coherence around dyskinetic movements^10^. Because 4 Hz trough PABST can decrease spike-field coherence, it might be particularly effective in dyskinesias.

Our work has several limitations. First, all real-time, closed-loop systems involve finite computation time and must account for latency by predicting dynamic brain conditions in the future. Our phase-latency is 13 ms. It is possible that with higher fidelity models of future brain state, phase accuracy will improve, enabling more effective brain stimulation^40^. Second, it is striking that we did not observe differences in LFP power but did find differences in spike-field coherence. These data suggest that PABST affected local neuronal populations, but not wider brain networks. It is possible that with stimulation of more D1-MSNs, or another circuit element, we could achieve more powerful effects. Third, while we did not observe reliable effects of PABST in sham animals or with phase-random stimulation, it is possible that additional mice, control groups and PD models may facilitate further interpretation of stimulation effects. Fourth, we note that 20 Hz stimulation of ChR2 does not have 100% spike efficiency^6,41,42^ and is far outside of the normal firing rate of D1-MSNs^43^. It is possible that other PABST strategies may have been more effective. Finally, our OptiTrack technology measures head movement, but there are many other aspects movements that our system does not capture^37,44^.

In summary, our work provides causal evidence of the relevance of phase to brain stimulation. Our phase-adaptive technology delivers low-latency stimulation which powerfully affects striatal spike-field coherence and increases movement velocity in dopamine-depleted mice. Future work will refine these methods and further refine PABST to deliver highly optimized and personalized brain stimulation with maximal efficacy.

## Supplementary Information

Supplementary Information 1.

Supplementary Video 1.

## Data Availability

All code and raw data are available at https://narayanan.lab.uiowa.edu.
