# Supplementary material for: Phase-adaptive brain stimulation of striatal D1 medium spiny neurons in dopamine-depleted mice
Source: Sci Rep. 2022 Dec 16;12:21780. doi: 10.1038/s41598-022-26347-z (PMC9758228; doi:10.1038/s41598-022-26347-z)
Supplement: Supplementary file 1 — Supplementary Information 1. [file 41598_2022_26347_MOESM1_ESM.docx]

**Supplementary Data**

**
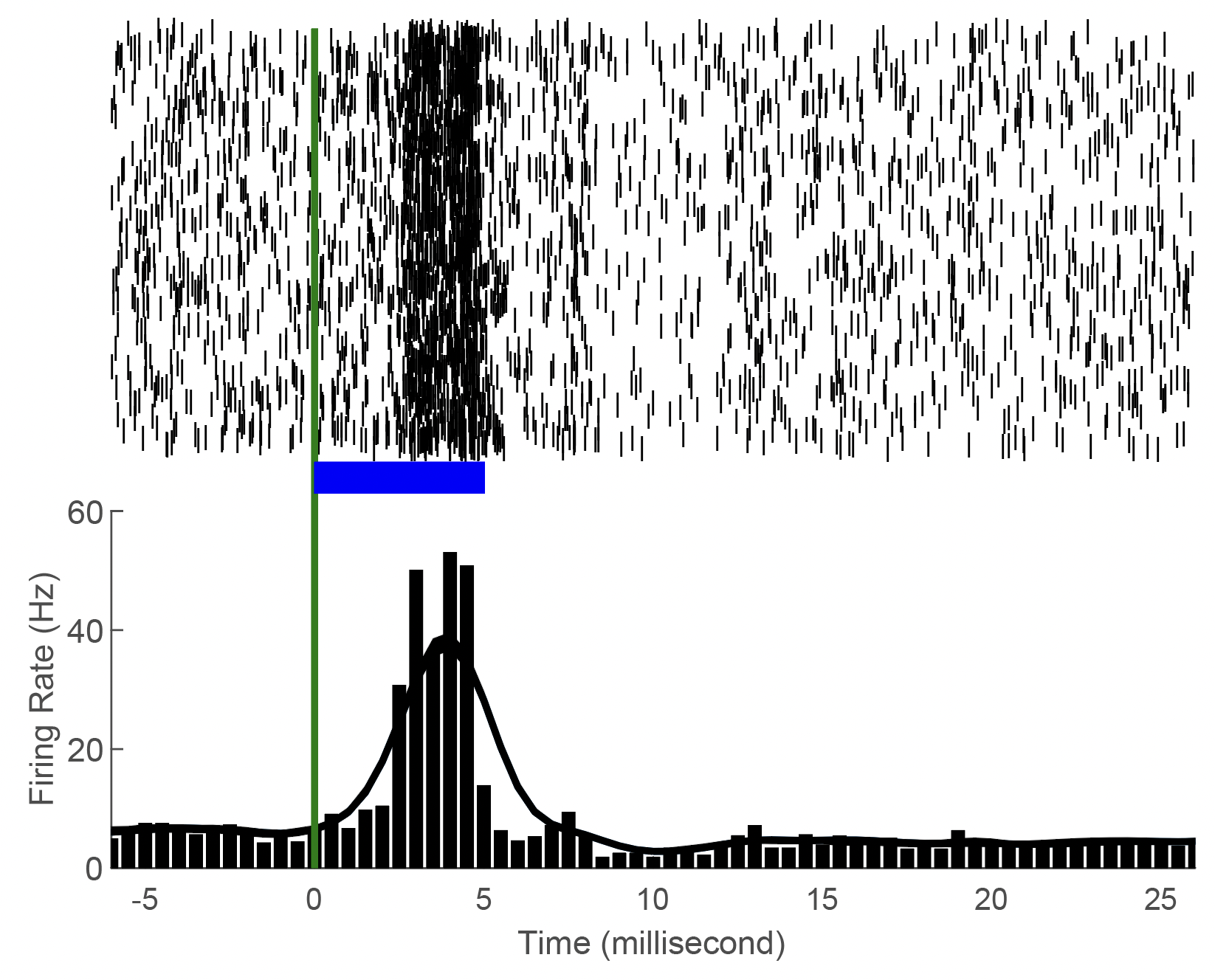
**

**Figure S1: An optogenetically-stimulated D1-MSN.** Top panel: peri-event raster of spiking activity in response to 5 millisecond pulse of 473-nm light (blue bar). Bottom panel: histogram of activity at from the top panel. The TTL pulse triggering the laser is represented by the vertical green line. This neuron markedly increased firing activity in response to laser pulses.


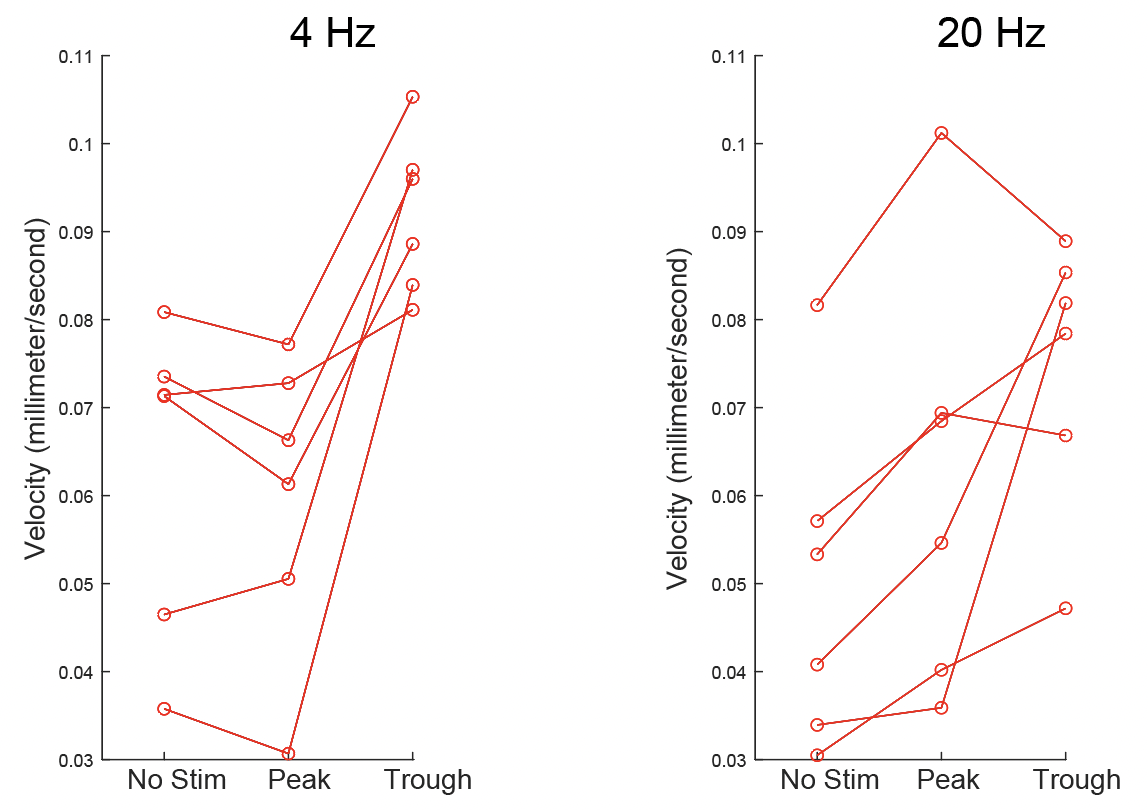


**Figure S2: Absolute velocity across animals.** Data from six animals for A) 4 Hz and B) 20 Hz PABST for No stimulation, Peak, and Trough trials.


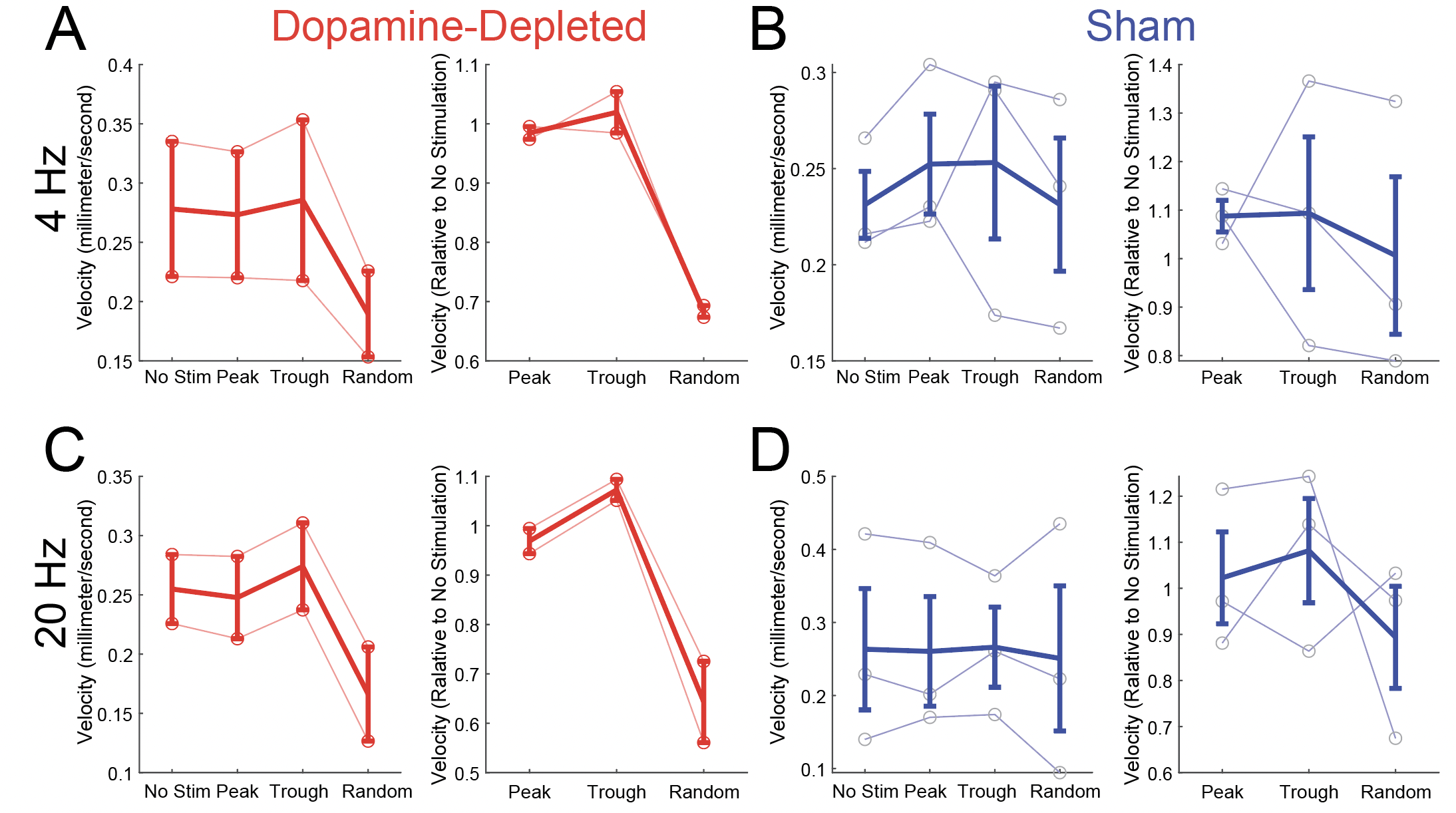


**Figure S3: Phase-random stimulation does not increase velocity.** A) In two separate dopamine-depleted animals, we compared phase-random stimulation at 4 Hz. In both dopamine-depleted animals, phase-random stimulation decreased velocity relative to no stimulation sessions. B) In three sham animals, we did not observe reliable effects of stimulation. C) In dopamine-depleted animals, 20 Hz phase-random stimulation also decreased velocity in 2/2 animals, but D) no reliable effects were seen in 3/3 sham animals.

­

**Supplementary Video:** 30-second video of a dopamine-depleted D1-Cre mice expressing ChR2 in the dorsal striatum. When the blue laser goes on for 4 Hz PABST, mouse head-movement increases, and the mouse starts moving around. We did not notice overt dyskinesias.
